# Favorable culture conditions for spermatogonial propagation in human and non-human primate primary testicular cell cultures: a systematic review and meta-analysis

**DOI:** 10.3389/fcell.2023.1330830

**Published:** 2024-01-08

**Authors:** Jillis van Maaren, Luis F. Alves, Madelon van Wely, Ans M. M. van Pelt, Callista L. Mulder

**Affiliations:** ^1^ Reproductive Biology Laboratory, Amsterdam UMC, University of Amsterdam, Amsterdam, Netherlands; ^2^ Amsterdam Reproduction and Development Research Institute, Amsterdam UMC, University of Amsterdam, Amsterdam, Netherlands; ^3^ Centre for Reproductive Medicine, Amsterdam UMC, University of Amsterdam, Amsterdam, Netherlands

**Keywords:** spermatogonial stem cells, fertility restoration, childhood cancer, spermatogonial propagation, culture methods

## Abstract

**Introduction:** Autologous transplantation of spermatogonial stem cells (SSCs) isolated from cryopreserved testicular biopsies obtained before oncological treatment could restore fertility in male childhood cancer survivors. There is a clear necessity for *in vitro* propagation of the limited SSCs from the testicular biopsy prior to transplantation due to limited numbers of spermatogonia in a cryopreserved testicular biopsy. Still, there is no consensus regarding their optimal culture method.

**Methods:** We performed a systematic review and meta-analysis of studies reporting primary testicular cell cultures of human and non-human primate origin through use of Pubmed, EMBASE, and Web of Science core collection databases. Of 760 records, we included 42 articles for qualitative and quantitative analysis. To quantify *in vitro* spermatogonial propagation, spermatogonial colony doubling time (CDT) was calculated, which measures the increase in the number of spermatogonial colonies over time. A generalized linear mixed model analysis was used to assess the statistical effect of various culture conditions on CDT.

**Results:** Our analysis indicates decreased CDTs, indicating faster spermatogonial propagation in cultures with a low culture temperature (32°C); with use of non-cellular matrices; use of StemPro-34 medium instead of DMEM; use of Knockout Serum Replacement; and when omitting additional growth factors in the culture medium.

**Discussion:** The use of various methods and markers to detect the presence of spermatogonia within the reported cultures could result in detection bias, thereby potentially influencing comparability between studies. However, through use of CDT in the quantitative analysis this bias was reduced. Our results provide insight into critical culture conditions to further optimize human spermatogonial propagation *in vitro*, and effectively propagate and utilize these cells in a future fertility restoration therapy and restore hope of biological fatherhood for childhood cancer survivors.

## Introduction

A potential novel fertility treatment for patients who will lose their reproductive potential due to gonadotoxic treatment at a pre-pubertal age, such as male childhood cancer survivors, is autotransplantation of *in vitro* propagated spermatogonial stem cells (SSCs) (Ridola et al., 2009; Hudson, 2010). For this therapy, a testicular biopsy is cryopreserved prior to cancer treatment to allow for fertility preservation of the SSCs present within the biopsy (Mirzapour et al., 2013; Masliukaite et al., 2016; Mulder et al., 2021). Transplantation of SSCs to restore spermatogenesis has been successful in multiple mammalian species, including non-human primates (Hermann et al., 2012; Takashima and Shinohara, 2018).

Since the establishment of the proof of principle of this SSC transplantation (Brinster and Avarbock, 1994; Brinster and Zimmermann, 1994), allotransplantation is considered as the gold standard of functional tests to show the presence of SSCs in the transplanted cell fraction, by demonstrating the cells’ capacity for migration to their natural niche and local colonization, followed by re-establishment of spermatogenesis. While auto- or allotransplantation with human material has not yet been performed, it is expected to yield similar results as success has also been obtained with allotransplantation of (uncultured) non-human primate testicular cells (Hermann et al., 2012). In the meantime, xenotransplantation of human cells to mouse models is considered as an equally functional test. Although full spermatogenesis cannot be achieved by human cells in the mouse testis, likely due to the niche differences in the testicular environment, the observable colonization and initial proliferation of these cells can be considered functional evidence of the presence of SSCs within the transplanted cell fraction (Nagano et al., 2002; Zhang et al., 2003).

The collection of testicular tissue for fertility preservation is considered to be safe for the patient when the biopsy is limited to 1 mL or an absolute maximum of 50% of total testicular volume (Uijldert et al., 2017). Though the number of SSCs collected from a human testicular biopsy may be expected to be relatively high compared to mice (Fayomi and Orwig, 2018; Sharma et al., 2019), the amount of SSCs needed to recolonize the entire, depleted, adult human testis is equally large. Furthermore, spermatogonial numbers may be reduced in childhood cancer patients prior to collection of the biopsy (Masliukaite et al., 2023). Therefore, *in vitro* propagation of SSCs is preferred prior to transplantation to increase colonization efficiency (Dobrinski et al., 1999; Nagano, 2003; Mirzapour et al., 2010; Mirzapour et al., 2015).

A successful culture system for murine SSCs was described for the first time in 2003 by Kanatsu-Shinohara et al. (2003). In this landmark publication, SSCs are maintained and propagated in specifically adapted StemPro-34 SFM medium within the presence of an exogenous feeder layer of mitotically inactivated mouse embryonic fibroblasts (MEF) for over 5 months. Identification of SSCs was confirmed by transplantation, which restored full spermatogenesis in infertile recipient mice and permitted generation of offspring (Kanatsu-Shinohara et al., 2003).

The first *in vitro* propagation system of human adult and pre-pubertal SSCs and subsequent successful xenotransplantation to mice was described 6 and 8 years after the results of Kanatsu-Shinohara et al., respectively, using similar culture conditions but lacking an exogenous feeder layer (Sadri-Ardekani et al., 2009; Sadri-Ardekani et al., 2011). In these human primary testicular cell cultures, the endogenous somatic cells originating from the patient’s testicular biopsy were used as an alternative to the exogenous and mitotically inactivated MEFs. However, due to the inherent high proliferative potential of some somatic cells, these cells tend to overgrow the cultures. Although the culture system employed by Sadri-Ardekani et al. was effective, as shown by successful human SSC colonization after xenotransplantation into mice testes, somatic overgrowth diluted the percentage of SSCs (Sadri-Ardekani et al., 2009; Sadri-Ardekani et al., 2011; Struijk et al., 2020b). Therefore, further optimization of this system is required prior to clinical implementation. However, unambiguous identification of SSCs remains complex.

Within cultures of primary testicular cells, spermatogonia morphologically appear as relatively small, round cells, initially floating within the culture medium or connected to the attached endogenous somatic cells, in time progressing to colonies with individually visible cells (Sadri-Ardekani et al., 2009; Mirzapour et al., 2017). The manner of verifying the presence of these SSCs in propagation cultures by use of protein or gene markers is hindered by the current lack of an unambiguously established SSC marker for human culture (Di Persio and Neuhaus, 2023). Therefore, a wide array of spermatogonial markers for PCR or immunofluorescence is used by researchers in an attempt to characterize the cultured cells, though these markers are often not specific for just one cell type and some markers have since been disputed due to their observed presence in somatic cells as well (Kossack et al., 2013; Struijk et al., 2020b). Conversely, instead of looking for specific markers researchers can also study specific behaviors of stem cells. In culture, SSCs form colonies and therefore an increase in colony numbers can be considered as presence of proliferative SSCs (Yeh et al., 2007).

Since the first reports of human primary testicular cell culture, many studies have followed using different culture methods with varying success. However, a concise review of the most optimal *in vitro* propagation system is currently lacking, thereby stalling clinical implementation of SSC transplantation. Therefore, this review aims to provide a systematic overview and meta-analysis of the current literature on human and non-human primate primary testicular cell cultures and to explore the effects of various culture conditions on spermatogonial colony doubling time *in vitro,* thus pressing ahead towards future therapeutic application within the clinic.

## Materials and methods

### Literature search

A literature search was performed in July 2022 within three databases: PubMed, Web of Science core collection, and EMBASE base + classic (OVID interface), to collect studies on the propagation of human and non-human primate SSCs. The full electronic search strategies for these databases can be found in Supplementary Note S1. The following exclusion criteria for studies were determined beforehand: no full text available or not written in English; only performed in immortalized cell lines; not involving cultures; focus on the differentiation of spermatogonia; data from species other than human and non-human primates; a complete lack of description of culture conditions; and use of fetal tissue, because of deviation from biology of the clinical target group at pre-pubertal age (Gaskell et al., 2004; Anderson et al., 2007; Sohni et al., 2019). Retrieved reviews were used as a source for citation searching, but the reviews themselves were excluded from qualitative and quantitative assessment. The review protocol was registered in the PROSPERO register of the National Institute for Health Research (registration ID: CRD42022342574).

### Screening process and risk of bias

The screening process, using COVIDENCE, involved at least two independent reviewers in each phase (L.F.A., C.L.M., A.M.M.v.P. and J.v.M.). After an initial screening of titles and abstracts, a full-text screening for eligibility was performed on the remaining articles to determine inclusion within the study. Risk of bias of included studies was assessed through a custom-made risk of bias tool. This tool was adapted from the Cochrane risk-of-bias-tool for randomized trials and the ROBINS-I assessment tool for non-randomized studies (Higgins et al., 2011; Sterne et al., 2016). Risk was assessed on confounding bias (tissue origin, sample allocation, tissue collection and handling); information bias (adequate description of culture method); performance bias (comparability of experimental conditions); detection bias (use of validated markers); measurement bias (adequate description of colony counts); analysis reporting bias (adequate description of statistical analyses); other bias (general statements). A detailed description of assessment at each level of risk can be found in Supplementary Table S1.

### Qualitative and quantitative analysis

All retrieved studies were thoroughly assessed for their main qualitative outcomes and their potential to be included in a quantitative analysis investigating the correlation of specific culture conditions on SSC propagation. Data extraction of included studies was performed by at least two independent researchers during each step (J.v.M., C.L.M. and A.M.M.v.P.). Ambiguous texts were discussed amongst the researchers and assumptions regarding study methods or outcomes were only made in cases where authors felt justified to do so. In cases where data remained unclear, the original authors of the paper in question were approached for further information.

Assessment of retrieved articles on their main quantitative outcomes involved total cell counts and colony counts during *in vitro* testicular cell cultures. Total colony counts were of specific interest to us because of the SSC origin of these colonies that is examined by this outcome, compared to total cell counts which are expected to also include somatic cell populations in cultures derived from testicular cell suspensions. Furthermore, the independence of any specific expression marker makes colony counts a more suitable method to compare findings across studies.

### Statistical analysis of quantitative outcomes

For quantitative analysis, colony doubling time (CDT) was calculated as an indication of SSC propagation and used to determine the effects of specific culture variables on this increase in the number of colonies in culture over time, using the formula: $CDT=t\;x\frac{\log2}{\left(logNtx-LogNt0\right)}$ , wherein t = culture duration between the time points of t0 and tx (days), Ntx = number of colonies at time point tx in culture and Nt0 = number of colonies at the time point in culture when colonies were first reported. Time point tx was determined by the last reported time point of culture, where colonies were reported (Hong et al., 2013; Gat et al., 2017).

In cases where colony counts at various time points were not specified within the text or tables, these numbers were determined from the graphs within the articles, when possible.

These data were then used to assess the correlation of the CDT with the specific culture conditions under which that certain CDT was obtained within a study. We could include the following conditions: enrichment methods prior to or during culture; use of feeders cells or non-cellular matrices; culture temperature; and medium composition, including the use of serum and addition of growth factors. Per condition, multiple categories were determined (e.g., temperature: 32°C, 34–35C° or 37°C). Each reported culture within an article, having resulted in a specific CDT, thus matched with only one category for each condition. Only studies without missing data points were included for analysis.

A generalized linear mixed model analysis, accounting for repeated measurements within each study, was used to calculate the association between the culture conditions and the CDTs of all included cultures. Results were expressed as mean differences with confidence intervals and data were analyzed using IBM SPSS Statistics for Windows, version 21. *p*-values <0.05 were considered to be statistically significant.

## Results

### Study retrieval

**FIGURE 1:**
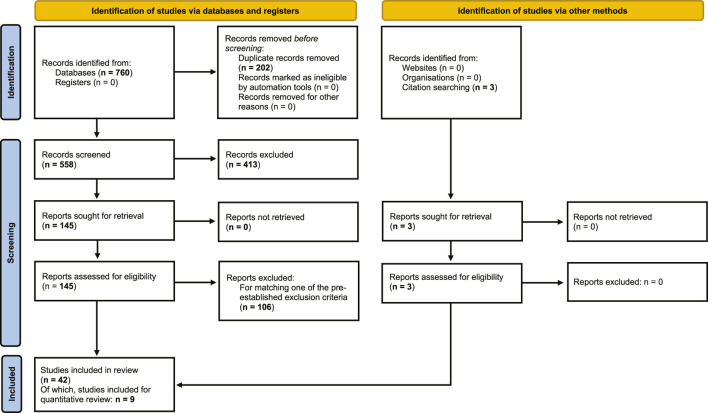
Flowchart of systematic review. Flowchart of search and selection of literature, to extract articles on human and non-human primate primary testicular cell cultures, for qualitative and quantitative assessment of outcomes of *in vitro* propagation of spermatogonia (based on PRISMA 2020 edition, Page et al., 2021).

From the literature searches in PubMed, Web of Science and EMBASE, a total of 760 articles were obtained, of which 202 duplicates were removed, resulting in 558 articles for screening. 413 records were excluded by screening of titles and abstracts based on exclusion criteria. Full-text screening was performed on the remaining 145 records, from which a further 106 reports were excluded, based on exclusion criteria. An additional 3 reports were found through citation searching, resulting in a total of 42 studies that were included in the qualitative synthesis of the review (Figure 1).

CDT could be determined in a total of 31 testicular cell cultures from 9 separate studies which were included in quantitative statistical analysis.

### Study characteristics

Out of 42 studies, 38 articles described research using human testicular tissue, and 4 articles described research using non-human primate testicular tissue, the latter including rhesus macaques, crab-eating macaques and marmoset monkeys. The research performed on human cells was done with testicular tissue of various sources. Adult tissues were collected from patients with a range of pathological backgrounds (brain-dead donors, n = 8; orchiectomy as part of prostate cancer therapy, n = 7; obstructive azoospermia (OA), n = 7; biopsy during vasectomy reversal surgery, n = 2; non-obstructive azoospermia (NOA), n = 15; orchiectomy due to testicular pathologies, n = 1; unknown, n = 1). Pre-pubertal tissues were collected prior to cancer treatments (n = 2) or during orchidopexy for uni- or bilateral cryptorchidism (n = 2).

Within the 42 studies using human and non-human primate testicular cultures, a multitude of variations within the culture protocol were applied that might influence spermatogonial propagation. These variables were explored in qualitative and quantitative analyses in this review. A customized risk of bias assessment was made to better suit the specifics of this review on culture protocols for spermatogonial propagation, and risk of bias was assessed on nine criteria for all 42 papers (Supplementary Figure S1). No articles were excluded based on the results of this risk of bias analysis.

### Qualitative data analysis

#### Enrichment methods

In the majority of the included studies, a similar approach is taken to isolate the testicular cells, including SSCs, from the testicular tissue. Subsequent enrichment methods are used as a means to improve the ratio between SSCs and other cell types before or during the testicular cell culture, through either the enrichment of spermatogonia or the removal of somatic cells (Lim et al., 2010). In many articles differential plating and/or subcultures are used, wherein the non-attached cell population or clusters containing spermatogonia are collected and further propagated, while the attached cells are discarded. Differential plating after isolation but prior to culture can result in a significant increase of putative spermatogonia in the floating cell fraction as detected by FACS and immunocytochemistry based on markers (Kim et al., 2017) as well as by formation of putative germ cell colonies after short-term culture (Kossack et al., 2013). The attached cell fraction is composed largely of cells with somatic cell morphology (Kossack et al., 2013). However, some overlap in cell populations is observed between the floating fraction and the attached cells after differential plating (Gat et al., 2017). Indeed, some SSCs are lost in the discarded attached cell fraction, as this fraction still possesses some colonization potential after xenotransplantation (Langenstroth et al., 2014). A sequential method with various coating substrates (gelatin, laminin) might enhance the efficiency of spermatogonial enrichment (Kim et al., 2017).

Enrichment through FACS or MACS could provide further enrichment, but requires antibodies against markers expressed on male undifferentiated germ cells (Nickkholgh et al., 2014; Struijk et al., 2020a; Struijk et al., 2020b). However, spermatogonial markers are often also expressed in somatic cells or differentiating germ cells (He et al., 2010). Therefore, markers can be lacking in specificity and further enrichment to reduce somatic cell overgrowth is often still necessary (Murdock et al., 2019). In various articles, the use of either FACS or MACS to select for *SSEA4*+ cells led to increased expression of spermatogonial markers in the cultured cell population and/or a larger number of colonies in the culture (Kokkinaki and Djourabtchi, 2011; Kim et al., 2017), or a higher number of colonies in the recipient testis after xenotransplantation (Kim et al., 2017), indicating enrichment of SSCs. However, most State 0 cells have low expression of *SSEA4*. State 0 germ cells are identified as the first of five distinct and progressive spermatogonial states as determined through singe cell sequencing analysis (Guo et al., 2018). Though several known early SSC markers are shared between State 0 and the following State 1, they each also show higher or even exclusive gene expression of other markers. State 2, conversely, shows expression of markers for spermatogonial differentiation. Though some plasticity between States 0 to 2 is likely (Guo et al., 2018), their separate suitability for culture and subsequent SSCT requires more in-depth study. In a study investigating the divergence of spermatogonial states in human, macaque and mouse, the earliest spermatogonial state (SPG1) showed enriched SSC activity based on xenotransplantation of human *TSPAN33+* sorted cells (Shami et al., 2020). However, the negative fraction also resulted in colonization, indicating that human SSCs are not restricted to this *TSPAN33*+ SPG1 population. Therefore, cell fractions for SSCT obtained by SSC enrichment based on a marker which is expressed in a limited number of cells in any of these states, such as *TSPAN33* and *SSEA4*, may exclude other transplantable SSC fractions. Additionally, some somatic cells exhibit a *SSEA4* ^dim^ phenotype, resulting in heterogeneous populations of testicular *SSEA4*+ cells after sorting (Zheng et al., 2014).

#### Feeder cells and non-cellular matrices

Feeder cells can provide mechanical support and adherence sites to spermatogonia and promote germ cell proliferation through various signaling molecules. In testicular cell cultures, feeder cells may be endogenously present within a cellular suspension obtained from the testicular biopsy or added from an exogenous source. Of the included articles in this review, a majority of cultures was performed without exogenous feeder cells (n = 27). The benefits of the presence of endogenous somatic cells within the testicular culture are illustrated by the study of Zahiri et al. who showed greater colony formation and diameter in cultures containing a testicular suspension obtained after enzymatic isolation without any further selection of cells, compared to enriched SSCs grown on uncoated plates, on exogenous feeder Sertoli cells or on nanofiber covered with laminin (Zahiri et al., 2020). However, when selecting highly specific cell populations from testicular tissue, the subsequent addition of exogenous feeder cells may be necessary to allow spermatogonial cell populations to attach and survive (Medrano et al., 2016; Gat et al., 2017).

In other cultures (n = 15) of the included articles, specifically isolated human or murine mitotic inactivated feeder cells were used within the culture to form a monolayer for the SSCs to attach to and support colony formation. Mouse embryonic fibroblasts (MEFs), human fetal placental fibroblasts and human fetal testicular fibroblasts were shown to provide inadequate support to maintain a culture of human SSCs (Smith et al., 2014; Murdock et al., 2019). However, other cells, such as *THY1*+ cells, endothelial cells and testicular multipotent stromal cells, have been demonstrated to provide an effective substrate to maintain SSCs (Eildermann et al., 2012; Smith et al., 2014; Bhang et al., 2018). In one study, the culture of testicular cells on a monolayer of Sertoli cells resulted in a higher total number of colonies than in a parallel control culture without additional feeder cells (Mirzapour et al., 2012). Even though feeder layers in general have relatively high confluence (Kanatsu-Shinohara et al., 2003), it was shown that low numbers of somatic cells (5,000 somatic cells) are able to support high numbers (up to 125.000 cells) of floating germ cells in establishing aggregates (Gat et al., 2017). To prevent overgrowth of feeder cells, mitotic inactivation by applying γ-radiation (Smith et al., 2014; Medrano et al., 2016) or mitomycin C treatment (Murdock et al., 2019; Yuan et al., 2022) may be applied.

To substitute the feeder layer which provides adherence sites for SSCs, non-cellular matrix coatings of the culture surface may be used. Piravar et al. showed that laminin coatings of culture dishes could replace the presence of feeder cells in a subculture of human germ line stem cell colonies during propagation for up to 6 weeks (Piravar et al., 2013). Similar beneficial effects of laminin were observed by Koruji et al. and by Jabari et al., the latter combining laminin with the use of agarose in a soft agar culture system (SACS) (Koruji et al., 2012; Jabari et al., 2020). A range of other decellularized extracellular matrix (ECM) substrates, including human testicular ECM and porcine testicular ECM, duodenal submucosa and urinary bladder ECM, were insufficient in supporting cultured human SSCs during culture for more than 2 weeks. However, it was shown that cultures on testicular tissue matrix from a homologous species performed slightly better than other tissues or tissues from another species (Murdock et al., 2019). The use of synthetic scaffolds has shown to support human spermatogonial propagation (Bashiri et al., 2022), but did not result in increased colony counts compared to cultures of spermatogonia on feeder cells originating from the testicular suspension (Borzouie et al., 2020; Zahiri et al., 2020). In contrast, hydrogel scaffolds composed with the use of platelet-rich plasma (PRP) showed increased SSC colony numbers compared to regular 2D cultures with and without the addition of PRP to the medium (Khadivi et al., 2020). Interestingly, these studies on the use of matrices in human primary testicular cell cultures are increasingly performed in recent years, illustrating the field’s search for alternatives to feeder layers in offering support structures for spermatogonial propagation.

#### Temperature

*In vitro* experiments in general are primarily performed at normal human body temperature (37°C). However, it is commonly known that the *in vivo* temperature of human testes is several degrees lower. Therefore, in theory, a culture of testicular cells might benefit from a lower culture temperature. Still, for primary testicular cell cultures an incubator temperature of 37°C is used in the majority of included articles (n = 22), with fewer cultures maintained at lower temperatures of 34°C–35°C (n = 9) and 32°C (n = 4). Culture temperature was not mentioned or unclear in seven studies.

Only one article included in this review directly investigated the effect of culture temperature on the number and proportion of simian spermatogonia. Through counting the number of *PLZF +* cells (representing undifferentiated spermatogonia) using immunocytochemistry, this study found increased total cell counts as well as increased spermatogonial numbers at 37°C compared to 34°C (Kim et al., 2017). No comparative studies were performed, however, with culture conditions including a temperature of 32°C.

#### Culture medium

To support and enhance the proliferation of any specific cell type *in vitro*, the composition of the culture medium is of great importance. Within the included articles, StemPro-34 SFM was used as culture medium in 24 studies, often supplemented according to that of mouse male germline stem cell (GS) cultures by Kanatsu-Shinohara et al. (2003); DMEM in 16 studies; and MEMα in 4 studies, the latter two supplemented with a variety of components. Basic medium was unclear in one study, and some studies used various media as separate culture conditions.

Direct comparison of the effect of various culture media on propagation of spermatogonia is scarce and results are contradictory, with studies reporting more pronounced colony growth in supplemented StemPro-34 SFM than in DMEM/F-12, or the other way around (Medrano et al., 2016; Gat et al., 2017). However, in the study by Medrano et al., only the StemPro-34 SFM culture was supplemented with growth factors, thereby potentially affecting the outcome (Medrano et al., 2016). As calculated by Gat et al. the population doubling time of testicular somatic cells was increased in DMEM/F-12 medium, compared to StemPro-34 SFM, and simultaneously a higher number of germ cell colonies (as described by the authors as aggregates of ≥10 cells) was found (Gat et al., 2017). Effects of basic medium on specific cell populations were seen by Kossack et al. as well, who compared supplemented Knockout DMEM and MEMα (Kossack et al., 2013). Cell cultures in MEMα medium showed increased expression of germ cell markers and decreased somatic cell markers, compared to cell cultures in Knockout DMEM Medium.

Studies on the effect of basic culture medium on culture outcome of spermatogonial propagation or somatic cell overgrowth are impeded by the unknown composition of various media, such as StemPro-34 SFM or KnockOut DMEM, and by the variety of supplements which are added by researchers. These supplements, including those described in the original protocol for propagation of mouse male GS cells (Kanatsu-Shinohara et al., 2003; Kanatsu-Shinohara et al., 2005), include not only sera and growth factors, but many nutrients and metabolic components as well. Their influence is illustrated by the observation that doubling the concentration of added supplements to MEMα (minimal essential medium, α modification) culture medium increased both the total numbers of testicular cells as well as the number of germ cells in culture (Kim et al., 2017).

Additionally, the use of various kinds and concentrations of serum could be of great influence on spermatogonial propagation. Alternatives to the commonly used fetal bovine serum (FBS) include the use of human serum or human platelet lysate, the latter of which has been shown to support *in vitro* proliferation of human SSCs (Dong et al., 2019a; Dong et al., 2019b). Differences may arise from using serum-based media compared to alternative, so-called “Knockout” or serum-free media (Wahab et al., 2016), which are often formulated to sustain specific cell populations and might lack certain components provided by serum. Within the reviewed articles, FBS was used in the vast majority of articles (n = 32) with a range of 0.25%–20% FBS, sometimes decreased in concentration during long-term culture (Akhondi et al., 2013). Higher amounts of FBS, usually 10%, were used most commonly in combination with DMEM. FBS in concentrations of ≤5% was generally combined with StemPro-34 SFM, which is on the market as a complete, serum-free medium and therefore likely contains enough components to decrease the necessary amount of added serum in human primary testicular cell cultures. Knockout Serum Replacement (KSR) was used in seven studies, ranging from 1% to 20%. Other serum-free media were used in another five studies, and use of serum was unknown in one study. No studies directly compared the effects of type or concentration of serum added to the medium on culture outcomes.

#### Growth factors

Growth factors, acting as signaling molecules to stimulate proliferation, are often added to a culture medium. Multiple growth factors are used within the reviewed articles, in varying concentrations and combinations. The growth factors and concentrations most often used are GDNF (10 ng/mL–100 ng/mL), bFGF (0.1 ng/mL–10 ng/mL), LIF (1,000–1,500 units/mL or 10 ng/mL) and EGF (20 ng/mL).

In conditions without exogenous feeder cells, the addition of these growth factors to the medium seems sufficient to allow for survival and propagation of testicular cells in long-term culture (Sadri-Ardekani et al., 2009; Sadri-Ardekani et al., 2011; Baert et al., 2015). The absence of growth factors in feeder-free cultures appears to be limiting or even detrimental to propagation of spermatogonia in culture (Koruji et al., 2012; Langenstroth et al., 2014). In monkeys, germ cell proliferation was promoted under a feeder-free culture condition involving all four growth factors (bFGF, LIF, EGF, and GDNF), compared to using only single growth factor (bFGF) or a limited subset (bFGF + either LIF, EGF or GDNF) (Kim et al., 2017). The growth factor bFGF, however, also strongly increased somatic cell proliferation (Eildermann et al., 2012). Still, a beneficial effect of bFGF on germ cell colony formation was seen in another study, when combined with supplementation of stem cell factor (SCF) in a 3D SACS culture system (Jabari et al., 2020). In this study, colony growth was further increased by the addition of EGF and laminin. The particular effect of SCF on SSC colonies could be disputed, however, as the presence of the SCF-receptor CKIT is generally agreed to be limited to differentiating spermatogonia, and absent on undifferentiated spermatogonial stem cells (Rossi et al., 2000).

In studies where an absence of growth factors was shown to lead to a high number of colonies (Mirzapour et al., 2012), exogenous feeder cells were used. The secretome of feeder cells such as endothelial cells (Bhang et al., 2018) or testicular somatic cells originating from the testicular cell suspension (Gat et al., 2017) most likely includes growth factors which may provide the spermatogonia with the necessary signals for propagation. However, additional supplementation with growth factors GDNF, EGF, LIF and bFGF may still be beneficial in cultures with feeder cells (Shiva et al., 2016), although this beneficial effect is not consistently seen, using only bFGF and LIF (Mirzapour et al., 2012). These studies indicate that the growth factor requirements of spermatogonia may differ when cultured with feeder cells or under feeder-free conditions.

Spermatogonial proliferation may be further promoted by additional medium compounds, such as PERIOSTIN protein (Li et al., 2021) or other yet unidentified compounds influencing the proliferative and signaling pathways of these cells (Guo et al., 2015). Similarly, suppressing or modifying pathways which normally suppress proliferation could yield similar proliferative effects, for instance through inhibition of transforming growth factor-beta (Moraveji et al., 2019) or by inhibiting pathways towards SSC differentiation (Tan et al., 2020).

### Quantitative analysis

A mixed model analysis was applied to the results of nine studies (with a total of 31 culture groups) where the spermatogonial CDT could be calculated based on reported colony counts or published graphs. Lower mean CDTs, as an indication of faster SSC propagation, were considered as preferential analysis outcomes. Of all included cultures, the average CDT was 21.22 ± 15.4 days (mean ± SD).

**FIGURE 2:**
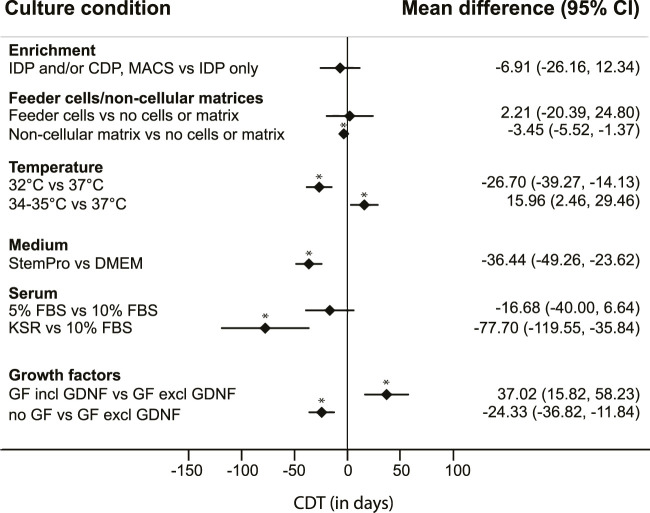
Mean differences in colony doubling time (CDT). Results of a mixed model analysis of a total of 9 studies, including 31 experimental groups, comparing mean CDT of six non-independent variables. Asterisks (*) indicate significant results, *p* < 0.05. IDP = differential plating of cells after isolation; CDP = differential plating of colonies during culture; MACS = magnetic-activated cell sorting. GF = growth factors.

Six culture conditions with 2-3 categories each could eventually be included in the analysis. The following conditions and corresponding categories were used: enrichment methods (differential plating of cells after isolation only; or differential plating of colonies during culture only or subculture combined with MACS or with differential plating of cells prior to culture); matrices (the use of feeder cells; use of non-cellular matrices; or neither feeder cells nor matrix); culture temperature (32°; 34°–35°; or 37°C); the type of medium used (StemPro-34 SFM or DMEM); the amount and type of serum added (≤5% FBS; 10% FBS; or KSR); and addition of growth factors (none; yes, including GDNF; or yes, but not including GDNF) (Figure 2). Additional categories were not feasible due to the limited number of included cultures.

From this quantitative analysis, the results regarding enrichment methods show a lower mean CDT in testicular cell cultures with either multiple enrichment methods or differential passaging of colonies during culture, compared to applying only differential plating at the start of the culture, although there was a large uncertainty around the estimate and these differences were not significant (Figure 2).

The presence of testicular somatic cells in the culture, still present after these enrichment methods, was not regarded as explicitly adding feeder cells. In most cultures with specifically added feeder cells, cellular populations described as Sertoli cells were used, obtained from the same testicular tissue of origin but separately processed. Cultures without added feeder cells or matrix were usually maintained on uncoated plastic dishes. Cultures using non-cellular matrices including laminin, Matrigel or SACS resulted in a significantly lower CDT, compared to CDT of cultures using neither feeder cells nor matrices (Figure 2).

Cultures that were maintained at a culture temperature of 32°C had a significantly lower mean CDT than cultures with a culture temperature of 37°C. However, the measured effect of temperature on CDT was not linear, as cultures maintained at 34°C–35°C had a significantly higher CDT than those with a culture temperature of 37°C (Figure 2).

Use of StemPro-34 SFM medium generated a significantly lower CDT than use of DMEM as basic culture medium (Figure 2). The StemPro-34 SFM media in these studies were supplemented largely according to recommendations by Kanatsu-Shinohara et al. (2003) with low percentages of FBS (1%–5%) and multiple growth factors including GNDF in all seven cases, whilst cultures performed in DMEM medium contained a wide variation in the absence or presence of specific growth factors, and generally a higher percentage of added FBS (5%–10%).

Serum itself as a variable showed effects on CDT as well, with KSR resulting in significantly lower CDT compared to cultures with 10% added FBS (Figure 2). During statistical modelling it was apparent that changes in data points within the serum parameter influenced the outcomes of the parameters “medium“ and “growth factors.” However, these changes never affected the direction of the measured effects.

To assess the effect of added growth factors on CDT, we divided the large variation of combinations of growth factors used in the included studies into larger categories: cultures without additional growth factors, and cultures with added growth factors to the culture medium. The latter category was further divided into addition of a set of growth factors which included GDNF, and those without GDNF. This distinction was added because GDNF is known to be a crucial factor in murine SSC proliferation *in vivo* and *in vitro* (Meng et al., 2000; Kubota et al., 2004; Takashima et al., 2015). Surprisingly, CDT analysis showed a significantly lower mean CDT in cultures without any added growth factors, compared to the other groups. Additionally, the CDT of cultures with added growth factors that did not include GDNF was significantly lower than the CDT of cultures with added growth factors including GDNF (Figure 2). The culture conditions across these groups were further qualitatively studied to assess differences amongst them. Nearly all (n = 18 out of 19) cultures without use of GDNF were maintained with 10% FBS in DMEM culture medium, and most of them (n = 17 out of 19) used feeder cells. In culture conditions where GDNF was added to the medium (either DMEM or StemPro-34 SFM), exogenous feeder cells were used in only 2 out of 12 cultures. The use of neither feeder cells nor matrix was more common (n = 4 out of 12), whilst the use of a non-cellular matrix was most prevalent (n = 6 out of 12 cultures) in this group.

## Discussion

This systematic review provides a unique contribution towards elucidation of the most optimal conditions to propagate spermatogonia in primary testicular cell cultures of human and non-human primates *in vitro*, a crucial step in the future application of SSC autotransplantation as a clinical fertility treatment. As established through qualitative and quantitative analysis, potential improvements for culture lie within a culture temperature of 32°C, the use of non-cellular matrices, the choice in culture medium and serum and the restriction of added growth factors.

The strength of our quantitative analysis rests within the performed calculation of CDT from studies that presented suitable quantitative data. The mixed model analysis that was performed on the CDTs provides a robust comparison of the CDTs of each of the described variables, as it takes into account their (partial) co-dependency. Under the assumption of Yeh et al. who showed correlations between *in vitro* colony numbers and number of SSCs (Yeh et al., 2007), this method of meta-analysis using CDT provides this field of research with a much needed and more systematic approach to the comparison of outcomes of primary testicular cell cultures between studies.

Some of our findings include culture conditions that have been implemented for years, such as the beneficial effect of differential plating of cells after isolation to reduce the amount of somatic cells at the start of culture, which are readily present within testicular biopsies and represent a heterogeneous population (Struijk et al., 2020b). However, the presence of feeder cells in culture may also contribute to establishment of the microenvironment to support propagation and differentiation of spermatogonia (van der Sanden et al., 2010). The use of patient-specific isolated testicular cells (e.g., endothelial cells, Sertoli cells or *THY1*+ cells) as feeder cells seems possible although, to prevent overgrowth, it may be necessary to inactivate proliferation of these cells. In cultures without feeder cells, the required secretome of feeder cells may be replaced by the addition of supplements, serum or growth factors to the medium, in combination with the presence of a specific matrix coating to support spermatogonial adherence. Our quantitative analysis showed that the use of non-cellular matrices in cultures generated a significantly lower mean CDT compared to those of cultures without any feeder cells or matrices. Laminin specifically might provide a suitable substrate for spermatogonial propagation within a testicular cell culture. From recent studies we have seen that the interest in use of 3D scaffolds in human primary testicular cell cultures has also increased (Borzouie et al., 2020; Jabari et al., 2020; Zahiri et al., 2020; Bashiri et al., 2022). Determining a suitable substrate to support cell-matrix interactions will be essential in controlling self-renewal of SSCs *in vitro* (van der Sanden et al., 2010). Further research into the *in vivo* composition of the SSC niche and its structural components will be of great benefit to mimic this *in vitro*.

Another important characteristic of the *in vivo* SSC environment is temperature. Data from the included studies suggest a shorter CDT in primary testicular cell cultures that are maintained at a temperature of 32°C, compared to 37°C. A lower optimal culture temperature would be in line with the *in vivo* physiology of the testis, the temperature of which is several degrees lower than the core body temperature in humans. This effect on temperature however does not seem linear, as the quantitative analysis showed a shorter CDT for cultures maintained at 37°C, compared to 34°C. This does support the findings by Kim et al. who found improved propagation of germ cells in simian cultures maintained at 37°C compared to 34°C (Kim et al., 2017). However, the study by Kim et al. did not include a culture temperature of 32°C. In humans *in vivo*, cryptorchid testes that are exposed to 37°C core body temperature are depleted of germ cells and are at greater risk of developing cancers (Ferguson and Agoulnik, 2013). Data on the effect of *in vitro* culture temperatures on spermatogonia are limited in humans and other species as well. It is known that heat shock treatment with temperatures over 40°C can significantly inhibit the proliferation of murine SSCs, by inducing cell cycle arrest in the S phase (Wang et al., 2019). In mice, cryptorchidism seems to induce dysregulation of somatic cells and SSCs, and depletion of the SSC pool through affecting self-renewal pathways (Ferguson et al., 2013). In line with this, porcine PTCC was reported to generate higher numbers of spermatogonia when cultured at 31°C, compared to 34°C and 37°C (Lee et al., 2013). As of yet, however, the number of studies directly comparing the effects of various culture temperatures on human spermatogonial propagation *in vitro* is too limited to draw any firm conclusions.

Studies on direct comparisons of the effect of basic culture media and their components on human and non-human primate testicular cell cultures are scarce as well. The culture medium used is of paramount importance in mimicking the human SSC microenvironment (Bardelli and Moccetti, 2017). Multiple authors use the medium formulation of StemPro-34 SFM as described by Kanatsu-Shinohara et al. (2003), which was successful in long-term maintenance of mouse male GS cell cultures. However, the optimal culture medium and supplements for culture of human SSCs are still unclear and may differ from murine requirements. Within the included studies the use of supplemented StemPro-34 SFM, DMEM (several varieties) and MEMα all seem to support colony formation to a certain extent, with StemPro-34 SFM showing decreased CDT compared to DMEM. However, there is a complex interplay between the medium, the added supplement and the paracrine effects of somatic cells present within the testicular cell culture as well. Within our analyses, the correlations between the culture medium and the added amounts of serum and growth factors are apparent. In cultures using DMEM as a basic medium, higher amounts of serum (most often FBS) are added in comparison to when StemPro-34 SFM is used. In mice, the addition of FBS to the culture medium has been reported to be detrimental to the proliferation of SSCs (Kubota et al., 2004), and serum-free culture systems are successfully used. Although our analysis showed a lower CDT in cultures using KSR than cultures using 10% FBS, such detrimental effects of higher amounts of FBS have to our knowledge not been found in human primary cultures before, and higher concentrations of FBS were in fact advantageous in the culture of immortalized human male germline stem cells (Hou et al., 2015).

Based on mouse studies, addition of the growth factor GDNF to the culture medium is generally assumed to be necessary to stimulate proliferation of male GS cells (Meng et al., 2000). Surprisingly, our study seems to indicate that in human PTCC the addition of GDNF to the culture medium is not beneficial, as a significantly higher CDT was found in cultures with GDNF. In fact, cultures without any added growth factors performed best in our quantitative analysis. Paracrine production of growth factors by somatic cells within the testicular cell suspension or feeder layer may compensate for lack thereof in the culture medium (Singh et al., 2017). Moreover, the addition of FBS may (partially) compensate lack of added growth factors, as can be concluded from reports showing good colony growth without any added growth factors and feeder cells (Mirzapour et al., 2012). Furthermore, the crucial role of GDNF in rodent PTCC may not be of similar importance in primate cultures. The receptor for GDNF, *GFRA1*, was found to be expressed in only low amounts in State 0 cells, which are indicated to be the earliest state of undifferentiated spermatogonia (Guo et al., 2018). In contrast, an analysis by Di Persio et al. found *GFRA1* to be reversely expressed to a higher extent in an earlier state and to be subsequently decreased later on (Di Persio et al., 2017). Therefore, the spermatogonial requirement for GDNF during human PTCC is currently unclear, but likely to vary between states of SSCs. Furthermore, a study by Tan et al. indicates that while GDNF can drive proliferation in cultures of human spermatogonia, its effect was not limited to primitive undifferentiated spermatogonia and was found to favour progenitor cells instead (Tan et al., 2020). Similarly, fibroblast growth factors (bFGF, syn. FGF2) could act differently in human spermatogonia than in rodent cells, potentially mediated through a divergence in functioning of the AKT pathway between mouse and human (Takashima et al., 2015; Tan et al., 2020). Finally, the addition of bFGF to human PTCC might especially stimulate the proliferation of somatic cells as well (Eildermann et al., 2012). Increased somatic cell proliferation could contribute to a decrease in germ cell proliferation through paracrine signaling or contact inhibition of cell growth (Leontieva et al., 2014). Such an increased somatic cell proliferation could explain why the CDT of cultures with added growth factors is longer than the CDT of cultures without any added growth factors, with otherwise similar culture conditions. In conclusion, the effect of the addition of growth factors may be influenced by culturing with or without feeder cells and the amount of serum (or serum replacement) in the culture medium. More thorough appraisal of the effect of individual growth factors on human PTCC is necessary, to elucidate their use in culture of SSCs for future SSCT.

Limitations of this review mostly relate to the large variety in the aims and designs of the included studies, thereby obstructing direct comparisons and limiting the number of studies suitable to be included in the quantitative analysis. Looking at our risk analysis, some additional parameters of the included studies may be considered as limitations of this review. Firstly, tissue origin between studies varied and may be an additional influence on culture success. Though tissues from various patient sources have been shown to result in spermatogonial propagation and colony formation, the number of colonies observed in culture may be affected by for instance the type of maturation arrest present in a patient’s testes (Nowroozi et al., 2011). Additionally, several studies lacked a histological diagnosis of the testicular biopsy of NOA patients and in these cases the use of a wider arrange of spermatogonial markers would be advisory, to exclude the potential of studying material of actual Sertoli cell only (SCO) patients. However, it could be assumed that in cultures of true testicular SCO material no SSC colonies would be formed and colony analysis therefore mitigates the bias on variation or lack of histological diagnoses. Ideally, more clinically relevant pre-pubertal tissues would be used for all future studies involving human primary testicular cell cultures, as the metabolic niche –and therefore *in vitro* requirements– of spermatogonia obtained from pre-pubertal and adult testes may vary (Voigt et al., 2023).

Furthermore, a certain risk of detection bias is created by the wide variety in type and amount of spermatogonial markers used within these studies, both within immunocytochemistry and PCR analyses. The use of CDT for quantitative analysis in our study however alleviates this obstacle by providing a more equal measurement of outcome, which is a strength of this study. Therefore, no articles were excluded based on the risk of bias analysis we performed. In calculating the CDT, it should be noted that the exponential growth phase is assessed but this excludes the initial lag phase of the culture.

In conclusion, based on these results, spermatogonial proliferation in human and non-human primate PTCC may benefit from the use of a non-cellular matrix, a lower culture temperature of 32°C, and use of a culture medium based on StemPro-34 SFM, with use of KSR, but without additional growth factors. In such a set-up, the endogenously present somatic cells in the cell suspension from the isolated biopsy are expected to contribute to the availability of mitogenic stimuli for the germ cells. Enrichment methods applied after cell isolation from a testicular biopsy should therefore not be too stringent in excluding these somatic cells.

More research specifically designed to assess the effect of one of these variables at the time is still required to elucidate the optimal method and medium composition for culture of human and non-human primate spermatogonia. The currently limited number of included studies in especially the quantitative analysis decreases the power of the analysis and a higher number of studies on spermatogonial propagation *in vitro* would contribute to the overall dataset available for these kind of comparative analyses. The use of experiments designed with a statistically determined, optimal set of conditions may facilitate the optimization process (Marinho et al., 2015). The use of a mixed model analysis like ours will be of an additional benefit to analyze outcomes of primary testicular cell cultures, as it accounts for repeated measurements within each study.

Although there are challenges we still have to face before patients can be offered an efficient and validated procedure to ensure SSC-based fertility restoration, the results of this systematic meta-analysis will provide support for efficient SSC propagation *in vitro* as the next step towards clinical implementation of SSC autotransplantation.

## Data Availability

The raw data supporting the conclusion of this article will be made available by the authors, without undue reservation.

## References

[B1] Akhondi M. M. Mohazzab A. Jeddi-Tehrani M. Sadeghi M. R. Eidi A. Khodadadi A. (2013). Propagation of human germ stem cells in long-term culture. Int. J. Reproductive Biomed. 11 (7), 551–558. PMC3941344 24639790

[B2] Anderson R. A. Fulton N. Cowan G. Coutts S. Saunders P. T. K. (2007). Conserved and divergent patterns of expression of DAZL, VASA and OCT4 in the germ cells of the human fetal ovary and testis. BMC Dev. Biol. 7, 136. 10.1186/1471-213X-7-136 18088417 PMC2211489

[B3] Baert Y. Braye A. Struijk R. B. Van Pelt A. M. M. Goossens E. (2015). Cryopreservation of testicular tissue before long-term testicular cell culture does not alter *in vitro* cell dynamics. Fertil. Steril. 104 (5), 1244–1252. 10.1016/j.fertnstert.2015.07.1134 26260199

[B4] Bardelli S. Moccetti M. (2017). Remodeling the human adult stem cell niche for regenerative medicine applications. Stem Cells Int. 2017, 6406025. 10.1155/2017/6406025 29090011 PMC5635271

[B5] Bashiri Z. Zahiri M. Allahyari H. Esmaeilzade B. (2022). Proliferation of human spermatogonial stem cells on optimized PCL/Gelatin nanofibrous scaffolds. Andrologia 54, e14380. 10.1111/and.14380 35083770

[B6] Bhang D. H. Kim B. J. Kim B. G. Schadler K. Baek K. H. Kim Y. H. (2018). Testicular endothelial cells are a critical population in the germline stem cell niche. Nat. Commun. 9 (1), 4379. 10.1038/s41467-018-06881-z 30348976 PMC6197186

[B7] Borzouie Z. Hekmatimoghaddam S. Jebali A. Aflatoonian B. (2020). The viability of human testis-derived cells on human serum albumin-based scaffold as an artificial male germ cell niche. Int. J. Fertil. Steril. 14 (2), 150–153. 10.22074/ijfs.2020.6086 32681628 PMC7382676

[B8] Brinster R. L. Avarbock M. R. (1994). Germline transmission of donor haplotype following spermatogonial transplantation. Proc. Natl. Acad. Sci. U. S. A. 91 (24), 11303–11307. 10.1073/pnas.91.24.11303 7972054 PMC45219

[B9] Brinster R. L. Zimmermann J. W. (1994). Spermatogenesis following male germ-cell transplantation. Proc. Natl. Acad. Sci. 91 (24), 11298–11302. 10.1073/pnas.91.24.11298 7972053 PMC45218

[B10] Di Persio S. Neuhaus N. (2023). Human spermatogonial stem cells and their niche in male (in)fertility: novel concepts from single-cell RNA-sequencing. Hum. Reprod. 38 (1), 1–13. 10.1093/humrep/deac245 36409992 PMC9825264

[B11] Di Persio S. Saracino R. Fera S. Muciaccia B. Esposito V. Boitani C. (2017). Spermatogonial kinetics in humans. Development 144 (19), 3430–3439. 10.1242/dev.150284 28827392

[B12] Dobrinski I. Ogawa T. Avarbock M. R. Brinster R. L. (1999). Computer assisted image analysis to assess colonization of recipient seminiferous tubules by spermatogonial stem cells from transgenic donor mice. Mol. Reproduction Dev. 53 (2), 142–148. 10.1002/(SICI)1098-2795(199906)53:2<142::AID-MRD3>3.0.CO;2-O 10331452

[B13] Dong L. Gul M. Hildorf S. Pors S. E. Kristensen S. G. Hoffmann E. R. (2019a). Xeno-free propagation of spermatogonial stem cells from infant boys. Int. J. Mol. Sci. 20 (21), 5390. 10.3390/ijms20215390 31671863 PMC6862004

[B14] Dong L. Kristensen S. G. Hildorf S. Gul M. Clasen-Linde E. Fedder J. (2019b). Propagation of spermatogonial stem cell-like cells from infant boys. Front. Physiology 10, 1155. 10.3389/fphys.2019.01155 PMC6761273 31607938

[B15] Eildermann K. Gromoll J. Behr R. (2012). Misleading and reliable markers to differentiate between primate testis-derived multipotent stromal cells and spermatogonia in culture. Hum. Reprod. 27 (6), 1754–1767. 10.1093/humrep/des091 22442249 PMC3357197

[B16] Fayomi A. P. Orwig K. E. (2018). Spermatogonial stem cells and spermatogenesis in mice, monkeys and men. Stem Cell Res. 29, 207–214. 10.1016/j.scr.2018.04.009 29730571 PMC6010318

[B17] Ferguson L. Agoulnik A. I. (2013). Testicular cancer and cryptorchidism. Front. Endocrinol. (Lausanne) 4, 32. 10.3389/fendo.2013.00032 23519268 PMC3602796

[B18] Ferguson L. How J. J. Agoulnik A. I. (2013). The fate of spermatogonial stem cells in the cryptorchid testes of RXFP2 deficient mice. PLOS ONE 8 (10), e77351. 10.1371/journal.pone.0077351 24098584 PMC3789668

[B19] Gaskell T. L. Esnal A. Robinson L. L. Anderson R. A. Saunders P. T. (2004). Immunohistochemical profiling of germ cells within the human fetal testis: identification of three subpopulations. Biol. Reprod. 71 (6), 2012–2021. 10.1095/biolreprod.104.028381 15317684

[B20] Gat I. Maghen L. Filice M. Wyse B. Zohni K. Jarvi K. (2017). Optimal culture conditions are critical for efficient expansion of human testicular somatic and germ cells *in vitro* . Fertil. Steril. 107 (3), 595–605. 10.1016/j.fertnstert.2016.12.028 28259258

[B21] Guo J. Grow E. J. Mlcochova H. Maher G. J. Lindskog C. Nie X. (2018). The adult human testis transcriptional cell atlas. Cell Res. 28 (12), 1141–1157. 10.1038/s41422-018-0099-2 30315278 PMC6274646

[B22] Guo Y. Liu L. Sun M. Hai Y. Li Z. He Z. (2015). Expansion and long-term culture of human spermatogonial stem cells via the activation of SMAD3 and AKT pathways. Exp. Biol. Med. 240 (8), 1112–1122. 10.1177/1535370215590822 PMC4935290 26088866

[B23] He Z. Kokkinaki M. Jiang J. Dobrinski I. Dym M. (2010). Isolation, characterization, and culture of human spermatogonia. Biol. reproduction 82 (2), 363–372. 10.1095/biolreprod.109.078550 PMC2809226 19846602

[B24] Hermann B. P. Sukhwani M. Winkler F. Pascarella J. N. Peters K. A. Sheng Y. (2012). Spermatogonial stem cell transplantation into rhesus testes regenerates spermatogenesis producing functional sperm. Cell Stem Cell 11 (5), 715–726. 10.1016/j.stem.2012.07.017 23122294 PMC3580057

[B25] Higgins J. P. Altman D. G. Gotzsche P. C. Juni P. Moher D. Oxman A. D. (2011). The Cochrane Collaboration's tool for assessing risk of bias in randomised trials. BMJ 343, d5928. 10.1136/bmj.d5928 22008217 PMC3196245

[B26] Hong S. H. Maghen L. Kenigsbcerg S. Teichert A. M. Rammeloo A. W. Shlush E. (2013). Ontogeny of human umbilical cord perivascular cells: molecular and fate potential changes during gestation. Stem Cells Dev. 22 (17), 2425–2439. 10.1089/scd.2012.0552 23557155

[B27] Hou J. Niu M. Liu L. Zhu Z. Wang X. Sun M. (2015). Establishment and characterization of human germline stem cell line with unlimited proliferation potentials and no tumor formation. Sci. Rep. 5, 16922. 10.1038/srep16922 26585066 PMC4653657

[B28] Hudson M. M. (2010). Reproductive outcomes for survivors of childhood cancer. Obstet. Gynecol. 116 (5), 1171–1183. 10.1097/AOG.0b013e3181f87c4b 20966703 PMC4729296

[B29] Jabari A. Sadighi Gilani M. A. Koruji M. Gholami K. Mohsenzadeh M. Rastegar T. (2020). Three-dimensional co-culture of human spermatogonial stem cells with Sertoli cells in soft agar culture system supplemented by growth factors and Laminin. Acta Histochem. 122 (5), 151572. 10.1016/j.acthis.2020.151572 32622422

[B30] Kanatsu-Shinohara M. Miki H. Inoue K. Ogonuki N. Toyokuni S. Ogura A. (2005). Long-term culture of mouse male germline stem cells under serum-or feeder-free conditions. Biol. Reprod. 72 (4), 985–991. 10.1095/biolreprod.104.036400 15601913

[B31] Kanatsu-Shinohara M. Ogonuki N. Inoue K. Miki H. Ogura A. Toyokuni S. (2003). Long-term proliferation in culture and germline transmission of mouse male germline stem cells. Biol. Reproduction 69 (2), 612–616. 10.1095/biolreprod.103.017012 12700182

[B32] Khadivi F. Koruji M. Akbari M. Jabari A. Talebi A. Ashouri Movassagh S. (2020). Application of platelet-rich plasma (PRP) improves self-renewal of human spermatogonial stem cells in two-dimensional and three-dimensional culture systems. Acta Histochem. 122 (8), 151627. 10.1016/j.acthis.2020.151627 33002788

[B33] Kim Y. H. Kang H. G. Kim B. J. Jung S. E. Karmakar P. C. Kim S. M. (2017). Enrichment and *in vitro* culture of spermatogonial stem cells from pre-pubertal monkey testes. Tissue Eng. Regen. Med. 14 (5), 557–566. 10.1007/s13770-017-0058-x 30603509 PMC6171622

[B34] Kokkinaki M. Djourabtchi A. Golestaneh N. (2011). Long-term culture of human SSEA-4 positive spermatogonial stem cells (SSCs). J. Stem Cell Res. Ther. 01 (S2). 10.4172/2157-7633.s2-003 PMC3898576 24466499

[B35] Koruji M. Shahverdi A. Janan A. Piryaei A. Lakpour M. R. Sedighi M. A. G. (2012). Proliferation of small number of human spermatogonial stem cells obtained from azoospermic patients. J. Assisted Reproduction Genet. 29 (9), 957–967. 10.1007/s10815-012-9817-8 PMC3463662 22735929

[B36] Kossack N. Terwort N. Wistuba J. Ehmcke J. Schlatt S. Schöler H. (2013). A combined approach facilitates the reliable detection of human spermatogonia *in vitro* . Hum. Reprod. 28 (11), 3012–3025. 10.1093/humrep/det336 24001715

[B37] Kubota H. Avarbock M. R. Brinster R. L. (2004). Growth factors essential for self-renewal and expansion of mouse spermatogonial stem cells. Proc. Natl. Acad. Sci. U. S. A. 101 (47), 16489–16494. 10.1073/pnas.0407063101 15520394 PMC534530

[B38] Langenstroth D. Kossack N. Westernströer B. Wistuba J. Behr R. Gromoll J. (2014). Separation of somatic and germ cells is required to establish primate spermatogonial cultures. Hum. Reprod. 29 (9), 2018–2031. 10.1093/humrep/deu157 24963164

[B39] Lee W.-Y. Park H.-J. Lee R. Lee K.-H. Kim Y.-H. Ryu B.-Y. (2013). Establishment and *in vitro* culture of porcine spermatogonial germ cells in low temperature culture conditions. Stem Cell Res. 11 (3), 1234–1249. 10.1016/j.scr.2013.08.008 24041805

[B40] Leontieva O. V. Demidenko Z. N. Blagosklonny M. V. (2014). Contact inhibition and high cell density deactivate the mammalian target of rapamycin pathway, thus suppressing the senescence program. Proc. Natl. Acad. Sci. 111 (24), 8832–8837. 10.1073/pnas.1405723111 24889617 PMC4066505

[B41] Li C. Cheng D. Xu P. Nie H. Zhang T. Pang X. (2021). POSTN promotes the proliferation of spermatogonial cells by activating the wnt/β-catenin signaling pathway. Reprod. Sci. 28 (10), 2906–2915. 10.1007/s43032-021-00596-1 33959891

[B42] Lim J. J. Sung S. Y. Kim H. J. Song S. H. Hong J. Y. Yoon T. K. (2010). Long-term proliferation and characterization of human spermatogonial stem cells obtained from obstructive and non-obstructive azoospermia under exogenous feeder-free culture conditions. Cell Prolif. 43 (4), 405–417. 10.1111/j.1365-2184.2010.00691.x 20590666 PMC6495878

[B43] Marinho P. A. Chailangkarn T. Muotri A. R. (2015). Systematic optimization of human pluripotent stem cells media using Design of Experiments. Sci. Rep. 5 (1), 9834. 10.1038/srep09834 25940691 PMC4419516

[B44] Masliukaite I. Hagen J. M. Jahnukainen K. Stukenborg J.-B. Repping S. van der Veen F. (2016). Establishing reference values for age-related spermatogonial quantity in prepubertal human testes: a systematic review and meta-analysis. Fertil. Steril. 106 (7), 1652–1657. 10.1016/j.fertnstert.2016.09.002 27717555

[B45] Masliukaite I. Ntemou E. Feijen E. A. M. van de Wetering M. Meissner A. Soufan A. T. (2023). Childhood cancer and hematological disorders negatively affect spermatogonial quantity at diagnosis: a retrospective study of a male fertility preservation cohort. Hum. Reprod. 38 (3), 359–370. 10.1093/humrep/dead004 36708005 PMC9977127

[B46] Medrano J. V. Rombaut C. Simon C. Pellicer A. Goossens E. (2016). Human spermatogonial stem cells display limited proliferation *in vitro* under mouse spermatogonial stem cell culture conditions. Fertil. Steril. 106 (6), 1539–1549. 10.1016/j.fertnstert.2016.07.1065 27490045

[B47] Meng X. Lindahl M. Hyvönen M. E. Parvinen M. de Rooij D. G. Hess M. W. (2000). Regulation of cell fate decision of undifferentiated spermatogonia by GDNF. Science 287 (5457), 1489–1493. 10.1126/science.287.5457.1489 10688798

[B48] Mirzapour T. Movahedin M. Ibrahim T. A. B. T. Haron A. W. Makoolati Z. Nowroozi M. (2010). Effect of donor cells concentration on colonization of human spermatogonial stem cells in recipient mouse testes. J. Biol. Sci. 10, 730–738. 10.3923/jbs.2010.730.738

[B49] Mirzapour T. Movahedin M. Koruji M. Nowroozi M. R. (2015). Xenotransplantation assessment: morphometric study of human spermatogonial stem cells in recipient mouse testes. Andrologia 47 (6), 626–633. 10.1111/and.12310 25209022

[B50] Mirzapour T. Movahedin M. Tengku Ibrahim T. A. Haron A. W. Nowroozi M. R. (2013). Evaluation of the effects of cryopreservation on viability, proliferation and colony formation of human spermatogonial stem cells *in vitro* culture. Andrologia 45 (1), 26–34. 10.1111/j.1439-0272.2012.01302.x 22621173

[B51] Mirzapour T. Movahedin M. Tengku Ibrahim T. A. Koruji M. Haron A. W. Nowroozi M. R. (2012). Effects of basic fibroblast growth factor and leukaemia inhibitory factor on proliferation and short-term culture of human spermatogonial stem cells. Andrologia 44 (1), 41–55. 10.1111/j.1439-0272.2010.01135.x 21806653

[B52] Mirzapour T. Tengku Ibrahim T. A. B. Movahedin M. Nowroozi M. R. (2017). Morphological and ultrastructural studies of human spermatogonial stem cells from patients with maturation arrest. Andrologia 49 (7), e12700–e12709. 10.1111/and.12700 27682317

[B53] Moraveji S. F. Esfandiari F. Taleahmad S. Nikeghbalian S. Sayahpour F. A. Masoudi N. S. (2019). Suppression of transforming growth factor-beta signaling enhances spermatogonial proliferation and spermatogenesis recovery following chemotherapy. Hum. Reprod. 34 (12), 2430–2442. 10.1093/humrep/dez196 31886487

[B54] Mulder R. L. Font-Gonzalez A. Green D. M. Loeffen E. A. H. Hudson M. M. Loonen J. (2021). Fertility preservation for male patients with childhood, adolescent, and young adult cancer: recommendations from the PanCareLIFE consortium and the international late effects of childhood cancer guideline harmonization group. Lancet Oncol. 22 (2), e57–e67. 10.1016/S1470-2045(20)30582-9 33539754

[B55] Murdock M. H. David S. Swinehart I. T. Reing J. E. Tran K. Gassei K. (2019). Human testis extracellular matrix enhances human spermatogonial stem cell survival *in vitro* . Tissue Eng. - Part A 25 (7-8), 663–676. 10.1089/ten.tea.2018.0147 30311859 PMC6482918

[B56] Nagano M. Patrizio P. Brinster R. L. (2002). Long-term survival of human spermatogonial stem cells in mouse testes. Fertil. Steril. 78 (6), 1225–1233. 10.1016/S0015-0282(02)04345-5 12477516

[B57] Nagano M. C. (2003). Homing efficiency and proliferation kinetics of male germ line stem cells following transplantation in mice. Biol. Reprod. 69 (2), 701–707. 10.1095/biolreprod.103.016352 12700185

[B58] Nickkholgh B. Mizrak S. C. Van Daalen S. K. M. Korver C. M. Sadri-Ardekani H. Repping S. (2014). Genetic and epigenetic stability of human spermatogonial stem cells during long-term culture. Fertil. Steril. 102 (6), 1700–1707. 10.1016/j.fertnstert.2014.08.022 25256932

[B59] Nowroozi M. R. Ahmadi H. Rafiian S. Mirzapour T. Movahedin M. (2011). *In vitro* colonization of human spermatogonia stem cells: effect of patient's clinical characteristics and testicular histologic findings. Urology 78 (5), 1075–1081. 10.1016/j.urology.2011.06.035 21908023

[B87] Page M. J. McKenzie J. E. Bossuyt P. M. Boutron I. Hoffmann T. C. Mulrow C. D. (2021). The PRISMA 2020 statement: an updated guideline for reporting systematic reviews. BMJ 372, n71. 10.1136/bmj.n71 33782057 PMC8005924

[B60] Piravar Z. Jeddi-Tehrani M. Sadeghi M. R. Mohazzab A. Eidi A. Akhondi M. M. (2013). *In vitro* culture of human testicular stem cells on feeder-free condition. J. Reproduction Infertil. 14 (1), 17–22. PMC3719359 23926556

[B61] Ridola V. Fawaz O. Aubier F. Bergeron C. de Vathaire F. Pichon F. (2009). Testicular function of survivors of childhood cancer: a comparative study between ifosfamide- and cyclophosphamide-based regimens. Eur. J. Cancer 45 (5), 814–818. 10.1016/j.ejca.2009.01.002 19216070

[B62] Rossi P. Sette C. Dolci S. Geremia R. (2000). Role of c-kit in mammalian spermatogenesis. J. Endocrinol. Investigation 23 (9), 609–615. 10.1007/BF03343784 11079457

[B63] Sadri-Ardekani H. Akhondi M. A. van der Veen F. Repping S. Van Pelt A. M. M. (2011). *In vitro* propagation of human prepubertal spermatogonial stem cells. Am. Med. Assoc. 305 (23), 2416. 10.1001/jama.2011.791 21673293

[B64] Sadri-Ardekani H. Mizrak S. C. Van Daalen S. K. M. de Winter-Korver C. M. Roepers-gajadien H. L. Koruji M. (2009). Propagation of human spermatogonial stem cells *in vitro* . Am. Med. Assoc. 302 (19), 2127–2134. 10.1001/jama.2009.1689 19920237

[B65] Shami A. N. Zheng X. Munyoki S. K. Ma Q. Manske S. L. Green C. D. (2020). Single-cell RNA sequencing of human, macaque, and mouse testes uncovers conserved and divergent features of mammalian spermatogenesis. Dev. Cell 54 (4), 529–547. 10.1016/j.devcel.2020.05.010 32504559 PMC7879256

[B66] Sharma S. Wistuba J. Pock T. Schlatt S. Neuhaus N. (2019). Spermatogonial stem cells: updates from specification to clinical relevance. Hum. Reprod. Update 25 (3), 275–297. 10.1093/humupd/dmz006 30810745

[B67] Shiva R. Ghasem S. Masoud H. Sadat K. L. Ali K. Reza D. M. (2016). Comparison of colony formation of human spermatogonial stem cells (SSCs) with and without collagen. J. Pak. Med. Assoc. 66 (3), 285–291. 26968278

[B68] Singh D. Paduch D. A. Schlegel P. N. Orwig K. E. Mielnik A. Bolyakov A. (2017). The production of glial cell line-derived neurotrophic factor by human sertoli cells is substantially reduced in sertoli cell-only testes. Hum. Reprod. 32 (5), 1108–1117. 10.1093/humrep/dex061 28369535 PMC6075567

[B69] Smith J. F. Yango P. Altman E. Choudhry S. Poelzl A. Zamah A. M. (2014). Testicular niche required for human spermatogonial stem cell expansion. Stem Cells Transl. Med. 3 (9), 1043–1054. 10.5966/sctm.2014-0045 25038247 PMC4149303

[B70] Sohni A. Tan K. Song H. W. Burow D. de Rooij D. G. Laurent L. (2019). The neonatal and adult human testis defined at the single-cell level. Cell Rep. 26 (6), 1501–1517. 10.1016/j.celrep.2019.01.045 30726734 PMC6402825

[B71] Sterne J. A. Hernán M. A. Reeves B. C. Savović J. Berkman N. D. Viswanathan M. (2016). ROBINS-I: a tool for assessing risk of bias in non-randomised studies of interventions. BMJ 355, i4919. 10.1136/bmj.i4919 27733354 PMC5062054

[B72] Struijk R. B. Dorssers L. C. J. Henneman P. Rijlaarsdam M. A. Venema A. Jongejan A. (2020a). Comparing genome-scale DNA methylation and CNV marks between adult human cultured ITGA6+ testicular cells and seminomas to assess *in vitro* genomic stability. PLoS One 15 (3), e0230253. 10.1371/journal.pone.0230253 32176716 PMC7075560

[B73] Struijk R. B. Mulder C. L. van Daalen S. K. M. de Winter-Korver C. M. Jongejan A. Repping S. (2020b). ITGA6+ human testicular cell populations acquire a mesenchymal rather than germ cell transcriptional signature during long-term culture. Int. J. Mol. Sci. 21 (21), 8269. 10.3390/ijms21218269 33158248 PMC7672582

[B74] Takashima S. Kanatsu-Shinohara M. Tanaka T. Morimoto H. Inoue K. Ogonuki N. (2015). Functional differences between GDNF-dependent and FGF2-dependent mouse spermatogonial stem cell self-renewal. Stem Cell Rep. 4 (3), 489–502. 10.1016/j.stemcr.2015.01.010 PMC4375941 25684228

[B75] Takashima S. Shinohara T. (2018). Culture and transplantation of spermatogonial stem cells. Stem Cell Res. 29, 46–55. 10.1016/j.scr.2018.03.006 29587218

[B76] Tan K. Song H.-W. Thompson M. Munyoki S. Sukhwani M. Hsieh T.-C. (2020). Transcriptome profiling reveals signaling conditions dictating human spermatogonia fate *in vitro* . Proc. Natl. Acad. Sci. 117 (30), 17832–17841. 10.1073/pnas.2000362117 32661178 PMC7395452

[B77] Uijldert M. Meißner A. de Melker A. A. van Pelt A. M. M. van de Wetering M. D. van Rijn R. R. (2017). Development of the testis in pre-pubertal boys with cancer after biopsy for fertility preservation. Hum. Reprod. 32 (12), 2366–2372. 10.1093/humrep/dex306 29040511

[B78] van der Sanden B. Dhobb M. Berger F. Wion D. (2010). Optimizing stem cell culture. J. Cell Biochem. 111 (4), 801–807. 10.1002/jcb.22847 20803548 PMC3348118

[B79] Voigt A. L. de Lima e Martins Lara N. Dobrinski I. (2023). Comparing the adult and pre-pubertal testis: metabolic transitions and the change in the spermatogonial stem cell metabolic microenvironment. Andrology 11, 1132–1146. 10.1111/andr.13397 36690000 PMC10363251

[B80] Wahab A. Y. A. Md Isa M. L. Ramli R. (2016). Spermatogonial stem cells protein identification in *in vitro* culture from non-obstructive azoospermia patient. Malays J. Med. Sci. 23 (3), 40–48. 27418868 PMC4934717

[B81] Wang J. Gao W.-J. Deng S.-L. Liu X. Jia H. Ma W.-Z. (2019). High temperature suppressed SSC self-renewal through S phase cell cycle arrest but not apoptosis. Stem Cell Res. Ther. 10, 227. 10.1186/s13287-019-1335-5 31358059 PMC6664773

[B82] Yeh J. R. Zhang X. Nagano M. C. (2007). Establishment of a short-term *in vitro* assay for mouse spermatogonial stem cells. Biol. Reproduction 77 (5), 897–904. 10.1095/biolreprod.107.063057 17687116

[B83] Yuan H. Sun J. Wang S. Xiang Z. Yang F. Yan Y. (2022). Primary culture of germ cells that portray stem cell characteristics and recipient preparation for autologous transplantation in the rhesus monkey. J. Cell Mol. Med. 26 (5), 1567–1578. 10.1111/jcmm.17197 35104031 PMC8899175

[B84] Zahiri M. Movahedin M. Mowla S. J. Noruzinia M. Koruji M. Nowroozi M. R. (2020). The epigenetic assessment of human spermatogenic cells derived from obstructive azoospermic patients in different culture systems. Urology J. 18 (2), 214–224. 10.22037/uj.v16i7.6092 33236339

[B85] Zhang X. Ebata K. T. Nagano M. C. (2003). Genetic analysis of the clonal origin of regenerating mouse spermatogenesis following transplantation. Biol. Reproduction 69 (6), 1872–1878. 10.1095/biolreprod.103.019273 12904317

[B86] Zheng Y. Thomas A. Schmidt C. M. Dann C. T. (2014). Quantitative detection of human spermatogonia for optimization of spermatogonial stem cell culture. Hum. Reprod. 29 (11), 2497–2511. 10.1093/humrep/deu232 25267789 PMC4191455

