# Supplementary material for: Favorable culture conditions for spermatogonial propagation in human and non-human primate primary testicular cell cultures: a systematic review and meta-analysis
Source: Front Cell Dev Biol. 2024 Jan 8;11:1330830. doi: 10.3389/fcell.2023.1330830 (PMC10800969; doi:10.3389/fcell.2023.1330830)
Supplement: Supplementary file 1 [file DataSheet1.PDF]

Supplementary Note 1
Systematic research query, related to the experimental procedures.
Date of search: July 12, 2022.

Pubmed
((Spermatogonia*[tiab] OR "Spermatogonium"[tiab] OR "SSC" [tiab] OR "SSCs" [tiab] OR ((Spermato*[tiab] OR "SSC"[tiab] or "SSCs"[tiab]) AND ("Type A"[tiab] OR "Type Ad"[tiab] OR "Type Ap"[tiab] OR "Ap"[tiab] OR "Ad"[tiab] OR "Type B"[tiab])) OR "Spermatogonia"[mh] OR "Adult Germline Stem Cells"[mh] OR "Spermatogenesis"[mh]) AND ("Proliferation"[tiab] OR "Proliferative"[tiab] OR "Propagation"[tiab] OR "proliferate"[tiab] OR "Propagate"[tiab] OR "Cell Proliferation"[mh]) AND ("culture*"[tiab] OR "culturing"[tiab] OR "vitro"[tiab] OR "invitro"[tiab] OR "medium*"[tiab] OR "coating*"[tiab] OR "matrix"[tiab] OR "scaffold*"[tiab] OR "2D"[tiab] OR "3D"[tiab] OR "Microfluidic*"[tiab] OR "Cell Culture Techniques"[mh] OR "Primary Cell Culture"[mh] OR "Culture Media"[mh]) AND (Human*[tiab] OR "Homo sapiens"[tiab] OR "Homini*"[tiab] OR "H. Sapiens"[tiab] OR “boys”[tiab] OR "Primate*"[tiab] OR "Monkey"[tiab] OR "ape*"[tiab] OR "prosimian"[tiab] OR "simian"[tiab] OR "Pan"[tiab] OR "chimpanzee*"[tiab] OR "Gorilla"[tiab] OR "Lemur*"[tiab] OR "orangutan*"[tiab] OR "gibbon*"[tiab] OR "tarsier*"[tiab] OR "marmoset*"[tiab] OR "baboon*"[tiab] OR "Macaque*"[tiab] OR "Macaca"[tiab] OR "Humans"[mh] OR "Primates"[mh])) NOT ("sclerosis"[tiab] OR "sheet-based"[tiab] OR "tumor"[ti] OR "cancer"[ti] OR "scleroderma"[tiab]) NOT (("mice"[ti] OR "murine"[ti] OR "rat*"[ti] OR "mouse"[ti] OR "rodent*"[ti] OR "cattle"[ti] OR "pig*"[ti] or "canine"[ti] or "domestic"[ti]) NOT ("Human*"[ti] OR "Homo sapiens"[ti] OR "H. Sapiens"[ti] OR "sapiens"[ti] OR “boys”[ti] OR "Primate*"[ti] OR Monkey[ti] OR "ape*"[ti] OR "prosimian"[ti] OR "simian"[ti] OR "Pan"[ti] OR "chimpanzee*"[ti] OR "Gorilla"[ti] OR "Lemur*"[ti] OR "orangutan*"[ti] OR "gibbon*"[ti] OR "tarsier*"[ti] OR "marmoset*"[ti] OR "baboon*"[ti] OR "Macaque*"[ti] OR "Macaca"[ti]))

Web of Science core collection
TS=(((Spermatogonia* OR "Spermatogonium" OR (("SSC" or "SSCs") AND ("type A" OR "Type Ad" OR "Type Ap" OR "Ap" OR "Ad" OR "Type B")) OR "SSC" OR "SSCs") AND ("Proliferation" OR "Proliferative" OR "Propagation" OR "Proliferate" OR "Propagate") AND ("culture*" OR "culturing" OR "vitro" OR "invitro" OR "medium" OR "coating" OR "matrix" OR "scaffold*" OR "2D" OR "3D" OR "Microfluidic*" OR "technique*") AND ("Human*" OR "Homo sapiens" OR "H. Sapiens" OR “boys” OR "Primate*" OR "Monkey" OR "ape*" OR "prosimian" OR "simian" OR "Pan" OR "chimpanzee*" OR "Gorilla" OR "Lemur*" OR "orangutan*" OR "gibbon*" OR "tarsier*" OR "marmoset*" OR "baboon*" OR "Macaque*" OR "Macaca")) NOT ("sclerosis" OR "sheet-based" OR "scleroderma" OR "mice" OR "murine*" OR "rat*" OR "mouse" OR "rodent*" OR "cattle" OR "pig*" OR "canine" OR "cat*" OR "domestic"))

EMBASE base+classic
((("Spermatogonia*" or "Spermatogonium" or (("Spermato*" or "SSC" or "SSCs") and ("Type A" or "Type Ad" or "Type Ap" or "Ap" or "Ad" or "Type B")) or "SSC" or "SSCs").ti,ab,kw. or exp spermatogonium/) and (("Proliferation" or "Proliferative" or "Propagation" or "proliferate" or "Propagate").ti,ab,kw. or exp cell proliferation/) and (("culture*" or "culturing" or "vitro" or "invitro" or "medium" or "coating" or "matrix" or "scaffold*" or "2D" or "3D" or "Microfluidic").ti,ab,kw. or exp cell culture/ or exp culture medium/ or exp cell culture technique/) and (("Human*" or "Homo sapiens" or "H. Sapiens" or "Primate*" or "Monkey" or "ape*" or "prosimian" or "simian" or "Pan" or "chimpanzee*" or "Gorilla" or "Lemur*" or "orangutan*" or "gibbon*" or "tarsier*" or "marmoset*" or "baboon*" or "Macaque*" or "Macaca").ti,ab,kw. or exp human/ or exp primate/ or exp prosimian/ or exp Haplorhini/ or exp Lorisidae/ or exp tarsier/)) not (("sclerosis" or "sheet-based" or "scleroderma").ti,ab. or ("cancer".ti. or "tumor") or (("mice" or "murine" or "rat*" or "mouse" or "rodent*" or "cattle" or "pig*" or "canine" or "domestic") not ("Human*" or "Homo sapiens" or "H. Sapiens" or "sapiens" or "Primate*" or Monkey or "ape*" or "prosimian" or "simian" or "Pan" or "chimpanzee*" or "Gorilla" or "Lemur*" or "orangutan*" or "gibbon*" or "tarsier*" or "marmoset*" or "baboon*" or "Macaque*" or "Macaca")).ti.)

Supplementary Table 1 - Description of customized risk of bias assessment of included studies per domain, topic and subject, as described within the experimental procedures of assessed reports. Colors in the risk column correspond to those used in Supplementary Figure 1 to indicate level of risk of bias (high/medium/low/not applicable).

Table legend:

SSC = spermatogonial stem cell

ICC = immunocytochemistry

(q)RT-PCR = (quantitative) reverse transcription - polymerase chain reaction

IgG = immunoglobulin G

**Domain**	**Topic**	**Subject**	**Assessment**	**Risk of bias**
**Prior to culture**	Confounding bias	Tissue origin	Tissue origin is adequately described; tissue contains unaffected SSCs.	High risk: tissue obtained from patients with non-obstructive azoospermia; orchiectomy due to testicular cancers; unknown origin and testicular histology.<br>Medium risk: tissue obtained at sex reassignment surgery after gender-affirming hormone treatment; healthy non-human primates; unknown origin with full spermatogenesis.<br>Low risk: tissue obtained from patients with obstructive azoospermia; vasectomy reversal surgery; orchiectomy due to prostate cancer; post-mortem organ donation; biopsy prior to cancer treatment (in cancer not affecting the testis).
		Sample allocation	Samples are adequately distributed amongst experimental conditions.	High risk: description is lacking or incomplete.<br>Medium risk: description is lacking or incomplete, but samples share a similar origin.<br>Low risk: adequate description of allocation.<br>Not applicable: only one condition included during culture.
		Tissue collection/handling	Tissue collection and handling (preservation, isolation) are adequately described.	High risk: description is lacking.<br>Medium risk: description is incomplete.<br>Low risk: description adequately provided.

**During culture**	Bias in classification of interventions – Information bias	Description of culture method	Data on culture method and medium is complete.	High risk: description is lacking.<br>Medium risk: description is incomplete.<br>Low risk: description adequately provided.
**After culture**	Bias due to deviations from intended interventions – Performance bias	Culture comparability	Culture conditions are sufficiently comparable, other than a single main variable (intervention).	High risk: comparison of culture outcomes is complicated by multiple conditions occurring simultaneously.<br>Medium risk: culture outcomes of experimental conditions are sufficiently comparable, despite minor difference in culture conditions.<br>Low risk: culture treatments and outcomes are comparable.<br>Not applicable: no experimental conditions included during culture.
	Bias in measurement of outcomes – Detection bias	Use of molecular markers for the presence of spermatogonia.	Use of multiple molecular markers per technique, with adequate controls.<br>(for ICC: use of isotype IgG.<br>(q)RT-PCR: replacement of cDNA by H20; omission of reverse transcriptase; mock transcription without RNA; use of housekeeping/reference gene essential.<br>(RT-)PCR: use of testicular tissue as positive control.)	High risk: use of a single molecular marker without adequate control; or no markers used at all to identify spermatogonia.<br>Medium risk: use of multiple molecular markers without adequate control; use of a single molecular marker with adequate control.<br>Low risk: use of multiple molecular markers with adequate controls.<br>Not applicable: only qualitative techniques without use of markers applied.

	Bias in measurement of outcomes – Measurement bias	Description of counting method	Technique (equipment) and methodology of cell or colony counts are described.	High risk: Counting methods are not described.<br>Medium risk: description of counting methods is unclear or incomplete.<br>Low risk: counting methods are adequately described.<br>Not applicable: only qualitative data.
	Bias in measurement of outcomes – Analysis reporting bias	Description of statistics	Appropriate description of statistical analyses of differences between culture conditions is provided.	High risk: Statistical analyses are not described.<br>Medium risk: description of statistical analyses is unclear or incomplete.<br>Low risk: statistical analyses are adequately described.<br>Not applicable: only qualitative data.
**Other bias, general statements:**<br>1. A statement of compliance to human/animal ethical rights is made.<br>2. A statement of potential conflicts of interest is made.				High risk: No statements are made regarding a license of animal experiments/human ethical rights and potential conflicts of interest.<br>Medium risk: A statement regarding either a license of animal experiments/human ethical rights or potential conflicts of interest is lacking; or, a relevant conflict of interest is reported.<br>Low risk: statements are made regarding both a license of animal experiments/human ethical rights and potential conflicts of interest.

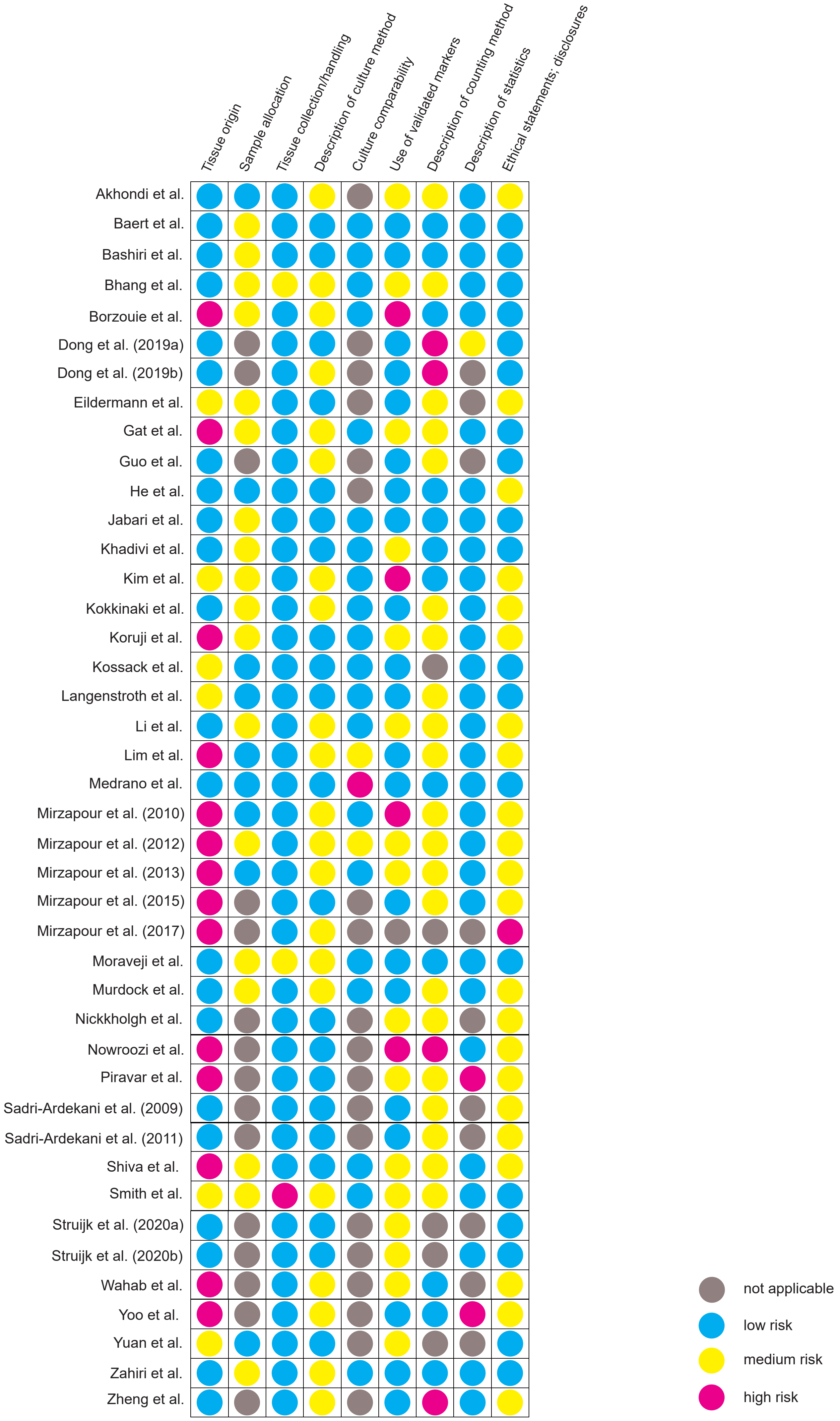

Supplementary Figure 1 - Assessment of risk of bias in included studies across 9 categories.
Relative risk is indicated by colors (grey: not applicable; cyan: low risk; yellow: medium risk; magenta: high risk).
